# Multivariate Frequency and Amplitude Estimation for Unevenly Sampled Data Using and Extending the Lomb–Scargle Method

**DOI:** 10.3390/s25216535

**Published:** 2025-10-23

**Authors:** Martin Seilmayer, Thomas Wondrak, Ferran Garcia

**Affiliations:** 1 Staatliche Studienakademie Bautzen, Duale Hochschule Sachsen, Löbauer Strasse 1, 02625 Bautzen, Germany; 2 Department of Magnetohydrodynamics, Helmholtz-Zentrum Dresden-Rossendorf, Bautzner Landstraße 400, D-01328 Dresden, Germany; t.wondrak@hzdr.de; 3 Department of Fluid Mechanics, Universitat Politècnica de Catalunya-BarcelonaTech, Av. Víctor Balaguer 1, Vilanova i la Geltrú, 08800 Barcelona, Spain; fernando.garcia-gonzalez@upc.edu

**Keywords:** multivariate data analysis, spectroscopic, uneven sampling

## Abstract

The Lomb–Scargle method (LSM) constitutes a robust method for frequency and amplitude estimation in cases where data exhibit irregular or sparse sampling. Conventional spectral analysis techniques, such as the discrete Fourier transform (FT) and wavelet transform, rely on orthogonal mode decomposition and are inherently constrained when applied to non-equidistant or fragmented datasets, leading to significant estimation biases. The classical LSM, originally formulated for univariate time series, provides a statistical estimator that does not assume a Fourier series representation. In this work, we extend the LSM to multivariate datasets by redefining the shifting parameter τ to preserve the orthogonality of trigonometric basis functions in $R^{n}$. This generalization enables simultaneous estimation of the frequency, phase, and amplitude vectors while maintaining the statistical advantages of the LSM, including consistency and noise robustness. We demonstrate its application to solar activity data, where sunspots serve as intrinsic markers of the solar dynamo process. These observations constitute a randomly sampled two-dimensional binary dataset, whose characteristic frequencies are identified and compared with the results of solar research. Additionally, the proposed method is applied to an ultrasound velocity profile measurement setup, yielding a three-dimensional velocity dataset with correlated missing values and significant temporal jitter. We derive confidence intervals for parameter estimation and conduct a comparative analysis with FT-based approaches.

## 1. Introduction

In numerous signal processing applications, the characterization of a physical process via its power spectrum or its amplitude and phase spectra is of fundamental importance. Typically, the recorded signal is sampled at uniform intervals over a finite observation period and stored as discrete sampled data. Spectral analysis serves multiple purposes, including the identification of characteristic frequencies for process recognition, the selection or attenuation of specific spectral components, and the precise estimation of the amplitude and phase at specified frequencies. Conventional approaches predominantly rely on the discrete Fourier transform (DFT) or its computationally efficient companion, the fast Fourier transform (FFT), both of which decompose the data into Fourier series coefficients from which the power spectrum can be derived [1,2]. These transformations are inherently invertible, computationally efficient, and straightforward to implement. However, their applicability is constrained by the requirement of equidistant and complete [1] sampling—limitations that pose challenges for data acquisition, in general. In practical applications, missing or corrupted data are often handled by substituting zero values, a common but problematic approach. While not the intention, zero values provide some sort of information and do not mean “not acquired”. Subsequently, zero values introduce a systematic error that leads to increased inaccuracies in frequency and amplitude estimation, ultimately affecting the reliability of parameter estimation. While the DFT naturally extends to higher dimensions—facilitating applications such as image reconstruction in magnetic resonance imaging [3], image filtering [4], and higher-order spectral filtering in spatiotemporal domains [5]—its performance deteriorates in the presence of non-uniform sampling. In ultrafast nuclear magnetic resonance (NMR) spectroscopy, non-uniform sampling enables the substantial acceleration of chemical analysis [6,7], albeit at the cost of increased missing data, reduced sensitivity, and signal attenuation. These limitations are inherent to DFT-based and similar techniques, as they exhibit reduced accuracy when applied to incomplete datasets with data gaps [8]. Although alternative methods, such as the “non-uniform Fourier transform” [9,10,11], attempt to address these issues, they remain reliant on classical orthogonal mode decomposition (e.g., FFT, DFT, wavelet transform) and thus inherit systematic errors, as discussed in Section 2.2. The recent publication by Wei and Yang [12] discusses the associated theory, complexity, and applications. They also compare different realizations of the non-uniform FFT.

The application of techniques based on Fourier series to signals with non-equidistant sampling is difficult, since it usually requires zero padding (replacing missing values with zeros) on a grid and, in the general case, resampling or interpolation of the data to a regular grid [13,14]. The effect of a gap-filling method was investigated extensively by Munteanu et al. [8]. They concluded that without a corrective measure, errors such as the amplitude minimization—which depends on the total gap density—cannot be avoided when FFT or DFT is applied. On the other hand, interpolation could be utilized to shift non-uniformly sampled data to a regular grid, such that the new interpolated data points comprise the signal information and a projection of the accompanying noise. In the general case, such distortions or noise are not band-limited, leading to biased estimates that consist of the local and aliased errors (e.g., noise, outliers, missing values) of the surrounding data.

Astrophysical data, for example, are affected by gaps, missing values, and uneven sampling. When ground-based radio telescopes are exploring areas in space, there may exist time intervals in which the antenna is not pointing towards the object of interest due to the rotation of the Earth. The result is an incomplete dataset. Non-uniform (random) sampling emerges; for example, the irregular appearance of objects, such as sunspots, is measured as a binary quality depending on the time and location. Section 5.3 provides a working example, which analyzes the frequencies and periods in latitude and time from the two-dimensional dataset of sunspot observations. Another technical example for random sampling (with highly variable sampling frequency) is asynchronous data acquisition in large sensor networks, which are used in Smart Homes, Industry 4.0, and automated driving; see Geneva et al. [15], Cadena et al. [16], Sudars [17]. Here, the time series provides missing values and data gaps originating from time periods in which either strong noise prevents the measurement or the source of the signal to be measured is not within the range of the sensor.

The Lomb–Scargle method (LSM) was developed especially for ground-based non-uniformly sampled one-dimensional data, from which the amplitude spectrum is calculated [18,19]. The main advantage of this method is that it can directly estimate the spectrum without the iterative optimization of trigonometric models, as discussed in Section 2.1. A fast version for one-dimensional signals was presented by Townsend [20] and Leroy [21]. As discussed in Mathias et al. [22], the computational complexity of estimating such a periodogram is significant. Consequently, even when using accelerated algorithms, refer to [20], the calculation remains a computationally intensive problem with a complexity of O(M·N), if *N* equals the number of samples and *M* the number of processed frequencies. Additionally, Zechmeister and Kürster [23] introduced a generalized Lomb–Scargle (GLS) method, which incorporates individual measurement errors for each sample and an extra constant term per frequency. Initially, all developments presented here will be based on the traditional Lomb–Scargle method. However, other related methods like GLS can be extended to multivariate analyses as well with the subsequently presented method. An implementation can be found in the corresponding R-Package (Version 2.0 from 2021) [24].

An extension of the LSM to two- or three-dimensional time series has not yet been presented. Especially for large multivariate datasets, this direct approach would be comparably faster than the present iterative procedures proposed in (Babu and Stoica [25], Chapter 9). The advantages of multivariate LSM are demonstrated in Section 5 by means of a 3D ultrasound flow profile measurement and the 2D analysis of sunspot time series data.

In the case of the analysis of flow profile measurements, which were obtained in an experiment investigating the magnetorotational instability in liquid metals [26], this method would be highly desirable. Due to the complex experimental setup and the weak signal-to-noise ratio, the flow profile measurements contain several time intervals in which distortions are dominant; see Seilmayer et al. [27]. These time intervals have to be rejected, leading to a time series with invalid (missing) data points, from which the multivariate version of the LSM is able to determine the parameters of the characteristic traveling wave.

In order to demonstrate the method and to motivate the basic idea of the multivariate version of the LSM, we compare it—in terms of the necessary conditions and error (noise) behavior—with the traditional orthogonal mode decomposition (OMD) with trigonometric basis functions, which is the essence of the classical Fourier transform. The LSM better fits the conditions of an arbitrary finite length of the sampling series in comparison with the DFT because the LSM reduces the error in the model parameter estimation. In contrast to the traditional approach, which was derived from a statistical point of view (see the appendix of Scargle [19]), the presented method is deduced from a technical point of view and focuses on its application. This leads to a slight change in the scaling of the model parameters, which is discussed in Section 4.2. However, the introduced procedure includes all the benefits, such as arbitrary sampling, fragmented data, and good noise rejection.

The starting point of this paper is the analysis of a continuous 1D signal s:R→R, which is composed of an arbitrary and finite set of individual frequency components $\omega_{i},0\leq i\leq M$. Without loss of generality, the band limitation is assumed, and there exists an upper maximum frequency $\omega_{\max}$ with $\omega_{i}<\omega_{\max},0\leq i\leq M$, which mimics an intrinsic low pass filter, characteristic of the measurement device. Since the measurement time is finite, the value of *s* is only known in the time interval $\left[0,T\right]$ with T∈R and T>0. The *m*-dimensional extension of this signal is $S:R^{m}\to R$. If such a continuous signal is sampled, the pair $\left({\hat{s}}_{i},t_{i}\right)\in R^{2},0\leq i\leq N-1$ represents the measured 1D value and the corresponding instant in time, whereas the pair $\left({\hat{S}}_{i},{\vec{t}}_{i}\right)\in R^{m+1},0\leq i\leq N-1$ represents a measured value and the *m*-dimensional location (${\vec{t}}_{i}$ space/time) for the *i*-th sample (*N* is the number of samples). All the methods presented herein were implemented in a package written in R and are published on CRAN [24]. The reader will find more elaborate examples in the package and in the Supplementary Materials

## 2. Mathematical Model and Comparison of OMD and LSM

In order to delineate the differences between the trigonometric OMD and LSM, we start with the basic model of a periodic signal as a sum of the signals of different frequencies $\omega_{k},0\leq k\leq M,$ with the corresponding amplitude $A_{k}\in R$ and phase shift $\varphi_{k}\in R$. The corresponding trigonometric model function

(1) $$\begin{array}{cc} y(t) & =\sum_{k=0}^{M}A_{k}\cos\left(\omega_{k}t+\varphi_{k}\right)=\sum_{k=0}^{M}(a_{k}\cos\left(\omega_{k}t\right)+b_{k}\sin\left(\omega_{k}t\right)) \end{array}$$

describes an infinite, stationary, and steady process y:R→R, with the coefficients $a_{k},b_{k}\in R$ for the defined frequency $\omega_{k}$ and the identities $A_{k}=\sqrt{a_{k}^{2}+b_{k}^{2}}$, as well as $\varphi_{k}=\tan^{-1}\left(b_{k}/a_{k}\right)$. Furthermore, the trigonometric model above consists of an arbitrary number $M\in N_{0}$ of frequency components. If the given signal s(t) is described by the defined model from Equation (1), the model misfit ϵ is given by

(2) ϵ(t)=s(t)−y(t).

Thus, the signal can now be described by inserting Equation (1) into Equation (2) and defining the misfit $\epsilon_{k}$ for each discrete frequency *k* with $\epsilon \left(t\right)=\sum_{k}\epsilon_{k}\left(t\right)$ as follows:

(3) $$\begin{array}{cc} s(t) & =\sum_{k=0}^{M}\left(a_{k}\cos\left(\omega_{k}t\right)+b_{k}\sin\left(\omega_{k}t\right)+\epsilon_{k}\left(t\right)\right). \end{array}$$

The defined misfit originates from the measurement uncertainties or parametric errors from $a_{k}$, $b_{k}$. In the general case, ϵ(t) can be any function or distribution. The challenge is to precisely determine the model parameters $a_{k}$ and $b_{k}$ for a given signal s(t) achieving minimal ϵ(t). This can be accomplished by one of the three methods described in the following sections: (i) least-square fit; (ii) orthogonal mode decomposition; (iii) Lomb–Scargle method (LSM).

### 2.1. Least-Square Fit

The optimal fit is reached by least-square fitting, resulting in a minimum ϵ, which was shown by Mathias et al. [22], Barning [28]. Since such procedures are iterative, the convergence of the algorithm might need a large number of function evaluations of Equation (3). Therefore, a direct version such as the LSM is preferable. The LSM becomes equivalent to a least-square fit of a sinusoidal model [18,28].

### 2.2. Trigonometric OMD

Generally, two functions f,g:R→R are said to be orthogonal on the interval $\left[a,b\right]\subset R,$ if the following condition holds; refer to Weisstein [29]:

(4) $$\int_{a}^{b}f\left(x\right)g\left(x\right)\;dx=0.$$

By selecting f(x)=sin(x) and g(x)=cos(x), the integration leads, by exploiting the identity $\cos\left(x\right)\sin\left(x\right)=\frac{1}{2}\sin\left(2x\right)$, to $\frac{1}{4}(\cos\left(2a\right)-\cos\left(2b\right))=0$, which is only zero if cos(2a)=cos(2b). This is true for any a∈R if the length of the interval [a,b] is a multiple of the period 2π, such that 2b=2a+2πk with $k\in N^{+}$. It is interesting to note that this interval can be shortened to one half of the period if cos(2a) is zero. By exploiting this feature of trigonometric functions, the individual model coefficients are calculated by multiplying the sine or the cosine to the measured data s(t) and integrating over all times as shown by (Cohen [1], Chapter 15):

(5) $$\begin{array}{cc} a_{k} & =\frac{2}{T}\int_{-\infty }^{\infty }s\left(t\right)\cos\left(\omega_{k}t\right)\;dt \end{array}$$

(6) $$\begin{array}{cc} b_{k} & =\frac{2}{T}\int_{-\infty }^{\infty }s\left(t\right)\sin\left(\omega_{k}t\right)\;dt. \end{array}$$

For a measured signal, the integration can only be performed over the interval $\left[0,T\right]$. It is obvious that an error is introduced if T≠2πn. In order to investigate the properties of the finite integration, we focus on the cosine term (Equation (5)), since the analysis of the sine term (Equation (6)) is similar. By setting the integral boundaries to the finite time interval and expressing the signal using Equation (3), the integral in Equation (5) can be written as

(7) $$\begin{array}{cc} \; & \int_{0}^{T}s\left(t\right)\cos\left(\omega_{k}t\right)dt=\\ \; & \int_{0}^{T}(a_{k}\cos^{2}\left(\omega_{k}t\right)+b_{k}\sin\left(\omega_{k}t\right)\cos\left(\omega_{k}t\right)+\epsilon_{k}\left(t\right)\cos\left(\omega_{k}t\right))\;\;dt. \end{array}$$

Using the identities $\cos^{2}\left(x\right)=\frac{1}{2}\left(1+\cos(2x)\right)$ and $\sin\left(x\right)\cos\left(x\right)=\frac{1}{2}\sin\left(2x\right)$, Equation (7) becomes

(8) $$\begin{array}{cc} \frac{2}{T}\int_{0}^{T}s\left(t\right)\cos\left(\omega_{k}t\right)\;dt & =a_{k}(1+\underset{\mathrm{truncation}\;\mathrm{error}\;\epsilon_{k}^{T}}{\underset{︸}{\frac{1}{T}\int_{0}^{T}(\cos\left(2\omega_{k}t\right)+\frac{b_{k}}{a_{k}}\sin\left(2\omega_{k}t\right))\;dt})}\\ \; & \;+\underset{\mathrm{random}\;\mathrm{error}\;\epsilon_{k}^{\mathrm{FS}}}{\underset{︸}{\frac{2}{T}\int_{0}^{T}\epsilon_{k}\left(t\right)\cos\left(\omega_{k}t\right)\;dt}}. \end{array}$$

The equation above indicates that the coefficient $a_{k}$ is effected by two errors $\epsilon_{k}^{T}$ (truncation) and $\epsilon_{k}^{\mathrm{FS}}$ (random), which might be nonzero for an arbitrary *T*. Thus, if both errors are neglected, the integral on the left hand side turns into an approximation of $a_{k}$. Taking the consideration about the orthogonality described in Equation (4) into account, $\epsilon_{k}^{T}$ only becomes zero if the integration time *T* is an integer multiple of $\pi /\omega_{k}$. This means for the Fourier series that a given integration time *T* (observation window) defines the lowest allowed frequency $\omega_{0}$. Furthermore, only multiples of this fundamental frequency $\omega_{k}=k\omega_{0}$ with k∈N are allowed in the Fourier series because $\epsilon_{k}^{T}=0$ in this case.

The technical realization of OMD is called “quadrature demodulation”, which is the discrete version of Equation (8) by replacing the integral over s(t) into a sum over the discrete sampled values ${\hat{s}}_{n},0\leq n\leq N-1$ and setting the truncation error to zero. For a time-discrete signal with *N* samples and equidistant sampling with a constant sampling period $T_{s}$, and $t_{n}=nT_{s}$, the parameter $a_{k}$ (corresponding to the frequency $\omega_{k}$) is approximated by

(9) $$a_{k}\approx \frac{2}{N}\sum_{n=0}^{N-1}{\hat{s}}_{n}\cos\left(\omega_{k}t_{n}\right).$$

**Figure 1:**
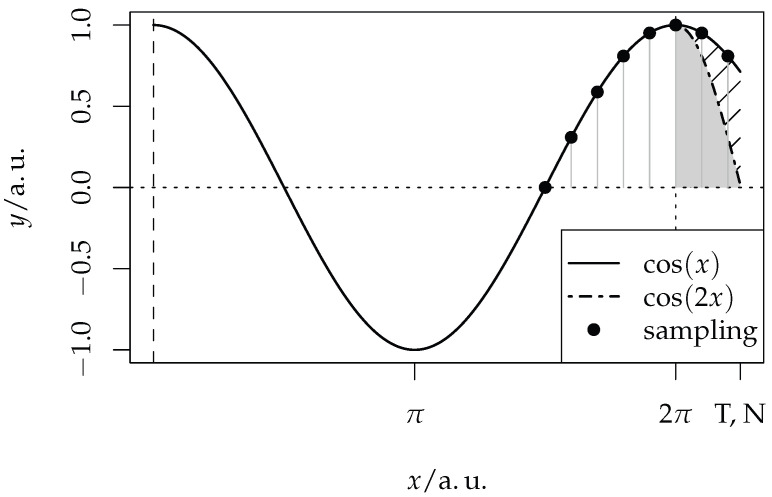
Example of the truncation error of a signal with the time period of 2π, which is generated by the last two samples. The value is indicated by the gray shaded area. The axes are scaled in arbitrary units (a.u.).

According to the previous considerations, the truncation error $\epsilon_{k}^{T}$ is larger than zero if the measurement range $T=NT_{s}$ is not exactly a multiple of $\pi /\omega_{k}$, as shown in Figure 1.

In this example, a signal with a time period of t=2π is sampled with N=23, $T_{s}=2\pi /20$. The total integration time is T=2π+1/5π, slightly longer than the period of the signal, which is indicated by the two additional sampling points after t=2π. The truncation error equals the gray shaded area.

Generally speaking, the sampling error in the discrete version can be estimated by

(10) $$\frac{2}{N}\sum_{n=0}^{N-1}{\hat{s}}_{n}\cos\left(\omega_{k}t_{n}\right)\approx a_{k}(1+\underset{\epsilon_{k}^{T}}{\underset{︸}{\frac{\Delta \varphi }{T}}})\pm \underset{\epsilon_{k}^{\mathrm{FS}}}{\underset{︸}{\Phi_{1-\alpha }\frac{2\sigma_{0}}{\sqrt{N}}}},$$

where $\Delta \varphi =\min_{i}\left(T-\pi i/\omega_{k}\right)$, i∈N. The random error is modeled by the α-quantile of the sampling error distribution $\Phi_{1-\alpha }$ related to the underlying process with its specific standard deviation $\sigma_{0}$ (e.g., JCGM [30]). This estimates the confidence interval of $a_{k}$ and $b_{k}$, respectively (see the Supplementary Material for details).

The above considerations lead to the following four statements, which are derived in detail in Section 2 of the Supplementary Materials: (i) the maximum absolute value of the truncation error is bounded $\left|\epsilon_{k}^{T}\right|\leq 0.2$ if $T\;≳\;\pi /\omega_{k}$, which is consistent with the experimental findings from Thompson and Tree [31]; (ii) $\epsilon_{k}^{T}$ is independent from the total number of samples *N* for a constant time *T*, which makes the OMD a non-consistent estimator for model parameters $a_{k}$ and $b_{k}$; (iii) by increasing the sample rate, the random error decreases by $O(N^{-0.5})$ but scales with twice the standard deviation of the noise distribution; and (iv) the DFT can be derived from Equation (10) via a restriction to equidistant sampling with constant $T_{s}$.

Concluding, the truncation error—which is an intrinsic feature of the OMD—produces a systematic deviation from the true value depending on the difference between the sampling time interval and the corresponding period of the frequency of interest. In contrast, the random error diminishes with an increasing sampling rate $T_{s}^{-1}=N/T$. A detailed discussion on this topic can be found in Thompson and Tree [31], Jerri [32], Shannon [33]. In the case of multivariate and randomly sampled data, the recent work of Al-Ani and Tarczynski [34] suggests two calculation schemes for estimating the Fourier transform in the general case. The continuous time Fourier transform estimation (similar to Equation (9)) takes samples with arbitrary spacing as the general approach, in contrast to the second proposed discrete time Fourier transform ($t_{n}=nT_{s}$ and $\omega_{k}=k\omega_{0}$) estimation scheme. Here, the data are projected onto a regular grid, which may provide the locations of missing data. Both schemes consider the sampling pattern $t_{n}$ and its power spectrum distribution function $p(t_{n})$. However, even for such sophisticated methods, the main conceptual drawbacks (see points (i) and (ii)) remain, which motivates the subsequent Lomb–Scargle method as a non-OMD method and its extension to multivariate data.

### 2.3. Lomb–Scargle Method

As described in the previous section, the disadvantage of the OMD is that the cosine and sine are not orthogonal on arbitrary intervals. By introducing an additional parameter τ∈R into Equation (4), we show that

(11) $$\int_{a}^{b}\cos\left(x-\tau \right)\sin\left(x-\tau \right)\;dx=0$$

holds for arbitrary intervals $\left[a,b\right]$. By utilizing the trigonometric identities $\cos^{2}\left(\phi \right)-\sin^{2}\left(\phi \right)=\cos\left(2\phi \right)$ and $\cos\left(\phi \right)\sin\left(\phi \right)=\frac{1}{2}\sin\left(2\phi \right)$, in order to remove the differences in the arguments, the integral can be transformed to

$$\int_{a}^{b}\left(\frac{1}{2}\sin\left(2x\right)\cos\left(2\tau \right)-\frac{1}{2}\sin\left(2\tau \right)\cos\left(2x\right)\right)\;dx=0.$$

If this integral is set to zero, the following condition holds

$$\cos\left(2\tau \right)\int_{a}^{b}\sin\left(2x\right)\;dx=\sin\left(2\tau \right)\int_{a}^{b}\cos\left(2x\right)\;dx,$$

which can be transformed to

(12) $$\frac{\int_{a}^{b}\sin\left(2x\right)\;dx}{\int_{a}^{b}\cos\left(2x\right)\;dx}=\tan\left(2\tau \right).$$

Therefore, from Equation (12), the parameter τ verifying Equation (11) may be found. In the case of equidistant sampling, the value of τ can be directly calculated from the integration boundaries by

$$\tau =\frac{b-a}{2}.$$

Based on this general consideration and in order to remove the truncation error, for each frequency $\omega_{k}$, the time shifting parameter $\tau_{k}\in R,0\leq k\leq M$ was introduced by Lomb and Scargle [18,19] into the model given in Equation (1); so,

(13) $$s\left(t\right)=\sum_{k=0}^{M}\left(a_{k}\cos\left(\omega_{k}\left(t-\tau_{k}\right)\right)+b_{k}\sin(\omega_{k}\left(t-\tau_{k}\right))+\epsilon_{k}\left(t\right)\right).$$

The parameter $\tau_{k}$ can be calculated by

(14) $$\tan\left(2\omega_{k}\tau_{k}\right)=\frac{\int_{a}^{b}\sin\left(2\omega_{k}t\right)\;dt}{\int_{a}^{b}\cos\left(2\omega_{k}t\right)\;dt},$$

similar to Equation (12). For the time-discrete version, the integrals transform into a sum, resulting in

(15) $$\tan\left(2\omega_{k}\tau_{k}\right)=\frac{\sum_{n=0}^{N-1}\sin\left(2\omega_{k}t_{n}\right)}{\sum_{n=0}^{N-1}\cos\left(2\omega_{k}t_{n}\right)}.$$

The parameters $a_{k}$ can be determined, beginning with Equation (7), but by factorizing them by $\cos^{2}\left(x\right)$, instead of expanding $\cos^{2}\left(x\right)$ to $\frac{1}{2}\left(1+\cos(2x)\right)$ as performed for Equation (8). In the following, the method is delineated for the discrete set ${\hat{s}}_{i}$, since it is applied to sampled data. These data are multiplied by $\cos(\omega_{k}\left(t_{n}-\tau_{k}\right))$, resulting in the following equation for a single frequency $\omega_{k}$:

(16) $$\sum_{n=0}^{N-1}{\hat{s}}_{n}\cos\left(\phi_{k}\right)=a_{k}\sum_{n=0}^{N-1}\cos^{2}\left(\phi_{k}\right)+b_{k}\underset{\mathrm{truncation}\;\mathrm{error}\;\epsilon_{k}^{T}}{\underset{︸}{\sum_{n=0}^{N-1}\sin\left(\phi_{k}\right)\cos\left(\phi_{k}\right)}}+\underset{\mathrm{random}\;\mathrm{error}\;\epsilon_{k}^{R}}{\underset{︸}{\sum_{n=0}^{N-1}\epsilon_{k}\left(t_{n}\right)\cos\left(\phi_{k}\right)}},$$

where $\phi_{k}=\omega_{k}\left(t_{n}-\tau_{k}\right)$. The sum of the terms $\cos\left(\phi_{k}\right)\sin\left(\phi_{k}\right)$ vanishes because of the proper selection of $\tau_{k}$, according to Equation (15). The sum of the terms $\epsilon_{k}\left(t_{n}\right)\cos\left(\phi_{k}\right)$ describes the modulated noise distribution function. The value of the parameters $a_{k}$ can be directly obtained by dividing by $\sum_{n=0}^{N-1}\cos^{2}\left(\phi_{k}\right)$. This gives

(17) $$\frac{\sum_{n=0}^{N-1}{\hat{s}}_{n}\cos\left(\phi_{k}\right)}{\sum_{n=0}^{N-1}\cos^{2}\left(\phi_{k}\right)}=a_{k}+\underset{\epsilon_{k}^{\mathrm{LS}}}{\underset{︸}{\frac{\sum_{n=0}^{N-1}\epsilon_{k}\left(t_{n}\right)\cos\left(\phi_{k}\right)}{\sum_{n=0}^{N-1}\cos^{2}\left(\phi_{k}\right)}}}.$$

The residual error term on the right-hand side, $\epsilon_{k}^{\mathrm{LS}}$, consists of a random distributed part divided by a sum over the square of the cosine. In contrast to the OMD, the estimation error of the parameter $a_{k}$ only depends on noise and is independent from the realization of sampling.

The next step is to determine $\epsilon_{k}^{\mathrm{LS}}$ in terms of a confidence interval with respect to α-quantile of the sampling error distribution $\Phi_{1-\alpha }$, as performed for the OMD in Equation  (10). Given a normal distributed error function with zero expectation value μ=0 and a standard deviation σ independent from time ($\epsilon_{k}↔N\left(0,\sigma \right)$), the error can be factorized (Parzen [35], Theorem 4A, p. 90), leading to

$$\begin{array}{cc} \epsilon_{k}^{\mathrm{LS}} & =\Phi_{1-\alpha }\frac{\sigma_{0}}{\sqrt{N}}\frac{\sum_{n=0}^{N-1}\cos\left(\phi_{k}\right)}{\sum_{n=0}^{N-1}\cos^{2}\left(\phi_{k}\right)}\leq \Phi_{1-\alpha }\frac{\sigma_{0}}{\sqrt{N}}e_{\max}, \end{array}$$

estimating the most probable limits of $\epsilon_{k}^{\mathrm{LS}}$. In the above equation, $e_{\max}$ does not depend on the frequency or the shifting parameter and is defined as

$$e_{\max}=\max_{\beta \in [0,2\pi ]}\left[\frac{\int_{0}^{\beta }\cos\left(\phi \right)\;d\phi }{\int_{0}^{\beta }\cos^{2}\left(\phi \right)\;d\phi }\right]=\max_{\beta \in [0,2\pi ]}\left[\frac{4\sin(\beta )}{\left(2\beta +\sin(2\beta )\right)}\right]=\frac{4}{\pi }.$$

The final parameter estimation gives

(18) $$a_{k}=\frac{\sum_{n=0}^{N-1}{\hat{s}}_{n}\cos\left(\omega_{k}\left(t_{n}-\tau_{k}\right)\right)}{\sum_{n=0}^{N-1}\cos^{2}\left(\omega_{k}\left(t_{n}-\tau_{k}\right)\right)}\pm \underset{\Delta a_{k}}{\underset{︸}{\frac{4}{\pi }\Phi_{1-\alpha }\frac{\sigma_{0}}{\sqrt{N}}}},$$

where the confidence interval $\Delta a_{k}=\frac{4}{\pi }\Phi_{1-\alpha }\frac{\sigma_{0}}{\sqrt{N}}$ converges to zero when increasing the number of samples *N*, for a fixed time interval. This property qualifies the LSM as a consistent estimator of the amplitude and phase [22]. In addition, the confidence interval $\Delta a_{k}$ for the LSM is smaller than the value given in Equation (10) for the OMD (this also occurs for the confidence interval of the parameters $b_{k}$, $\Delta b_{k}=\Delta a_{k}$). We notice that the definition of $a_{k}$ given in Equation (18) is slightly different from the original definition given by Lomb [18], but both definitions converge for large *N* (see the Supplementary Materials).

The confidence interval for the amplitude, $A_{k}$, can be deduced by propagating the error $\Delta a_{k}$:

$$\begin{array}{cc} \Delta A_{k} & =|\frac{\partial A_{k}}{\partial a_{k}}|\Delta a_{k}+|\frac{\partial A_{k}}{\partial b_{k}}|\Delta b_{k}=\frac{4}{\pi }\Phi_{1-\alpha }\sqrt{\frac{2}{N}}\sigma_{0}. \end{array}$$

In a similar way, the confidence interval for the phase $\varphi_{k}$ can be defined by

$$\begin{array}{cc} \varphi_{k} & =\tan^{-1}\left(\frac{b_{k}}{a_{k}}\right)\pm \Delta \varphi =\tan^{-1}\left(\frac{b_{k}}{a_{k}}\right)\pm \frac{4}{\pi }\Phi_{1-\alpha }\sqrt{\frac{2}{N}}\frac{\sigma_{0}}{A_{k}}. \end{array}$$

It is interesting to note that the confidence interval for the phase $\varphi_{k}$ decreases for increasing amplitude.

## 3. Spectrum from the Lomb–Scargle Method

In order to determine the spectrum with the LSM, we assume the recorded signal is described by many frequencies. The most significant frequency is then represented by a peak in the frequency spectrum of a certain width and height. The width is determined by the frequency resolution Δf, which equals 1/T. From this point of view, the precision of a frequency estimation changes only with the observation length *T* and seems to be independent from the number of samples *N* and the signal quality.

The quality Σ is measured by a signal-to-noise ratio-like expression

(19) $$\begin{array}{cc} \Sigma & =\sqrt{\frac{1}{N}\sum_{n=0}^{N-1}\frac{{\left(s_{n}-y_{n}\right)}^{2}}{\sigma_{n}^{2}}}\approx \frac{\sqrt{\sum_{l}A_{l}^{2}}}{\sqrt{\frac{1}{N}\sum_{n=0}^{N-1}{\left(\epsilon (t_{n})\right)}^{2}}}, \end{array}$$

with $A_{l}$ counting the *significant* amplitudes. In Equation (19), $y_{n}$ is the fitted model, $\sigma_{n}$ is the uncertainty per sample, with $\sigma =\sqrt{\frac{1}{N}\sum_{n=0}^{N-1}\sigma_{n}^{2}}$, and $\epsilon (t_{n})$ is the noise per sample. Following VanderPlas [36], we apply Bayesian statistics and assume that every peak is Gaussian-shaped, i.e., $e^{P(f_{\max}\pm \Delta f)}\propto e^{-\Delta f^{2}/\left(2\sigma_{f}^{2}\right)}$. It follows that a significant peak $A_{\max}^{2}=A^{2}\left(f_{\max}\right)$ appears at $f_{\max}$, in such a way that $A_{\max}^{2}/2=A^{2}\left(f_{\max}\pm \Delta f\right)$ is valid. Here, $A^{2}$ is related to the power spectral density $P\left(f\right)\propto a_{k}^{2}+b_{k}^{2}$. The frequency uncertainty (or standard deviation) is then given by

$$\sigma_{f}\approx \Delta f\sqrt{\frac{2}{N\Sigma^{2}}},$$

so that a significant peak is located in the interval $f_{\max}\pm \sigma_{f}$. This approach suggests that increasing the number of samples in a fixed interval *T* enhances the precision of $f_{\max}$ by reducing $\sigma_{f}.$ However, if the original signal contains two frequencies with a distance in the range of 1/T, it cannot be excluded, even for the LSM, that these peaks merge together into a single peak with small $\sigma_{f}$, according to Kovács [37].

As a statistical measure, the probability $P(P_{k}>P_{0})$ states that there is no peak $P_{k}$ larger than a reference value $P_{0}$ of the best fit. From here, the statistical significance of a single frequency $\omega_{k}$ can be deduced as the so-called false alarm probability (FAP) with

(20) $$\mathrm{FAP}=\left\{\begin{array}{cc} 1-{\left(1-P(P_{k}>P_{0})\right)}^{M}\;, & \mathrm{if}\;P(P_{k}>P_{0})\approx 1\\ MP(P_{k}>P_{0}), & \mathrm{if}\;P(P_{k}>P_{0})\ll 1 \end{array}\right.,$$

where *M* denotes the number of independent (fundamental) frequencies present in the signal. Horne and Baliunas [38] carried out an extensive study about the number of independent frequencies (and the maximum detectable frequency). They found an empirical approximation

$$M=-6.362+1.193N+0.00098N^{2},$$

which is a compromise between the conservative N/2 and the artificially large minimal distance value. A detailed discussion on FAP and the independent frequencies can be found in the studies by Baluev [39,40,41]. Furthermore, the Supplemetary Materials provide a detailed discussion on calculating power spectral density (PSD), $P_{k}=\frac{N}{4\sigma_{0}^{2}}\left(a_{k}^{2}+b_{k}^{2}\right)$, and its standardized companion $p_{k}\in \left[0,1\right]$ according to Zechmeister and Kürster [23] and Hocke [42].

Summarizing the properties of the LSM: (i) there is no truncation error $\epsilon_{T}=0$; (ii) the LSM provides better noise rejection compared to the OMD, $\epsilon^{\mathrm{LS}}<\epsilon^{\mathrm{FS}}$; (iii) the specific sampling pattern influences the spectrum calculated using LSM or another OMD-based method. The reversal of this effect is beyond the scope of this work.

## 4. The Multivariate Lomb–Scargle Method

A signal *S* depending on *m*-independent variables represents a function $S\left(\vec{t}\right):R^{m}\to R$ with the input vector described by $\vec{t}=\left[t_{1},t_{2},\ldots ,t_{m}\right]$. The model function for a multivariate LSM is gained by replacing the scalar arguments of the cosine and sine of the univariate model function of Equation (13) with vectors, resulting in

$$Y\left(\vec{t}\right)=\sum_{k=0}^{M}\left(a_{k}\cos\left({\vec{\omega }}_{k}\cdot \left(\vec{t}-{\vec{\tau }}_{k}\right)\right)+b_{k}\sin\left({\vec{\omega }}_{k}\cdot \left(\vec{t}-{\vec{\tau }}_{k}\right)\right)\right).$$

In this case, the shifting parameter ${\vec{\tau }}_{k}\in$$R^{m},0\leq k\leq M$ is a vector and, in principle, hard to calculate. However, if the argument of the cosine is expanded, it is obvious that the scalar product ${\vec{\omega }}_{k}\cdot {\vec{\tau }}_{k}\in R$ does not depend on the time; thus, the cosine argument can be written as ${\vec{\omega }}_{k}\cdot \vec{t}-\tau_{k}^{*}$ with $\tau_{k}^{*}={\vec{\omega }}_{k}\cdot {\vec{\tau }}_{k}$. The determination of $\tau_{k}^{*}$ is similar to that of τ shown in Equation (14); however, there are some differences in the equations, since the phase, instead of the time coordinate, is shifted now. This determination is described in the following.

### 4.1. Derivation of the Shifting Parameter

This section shows that shifting the phase, instead of the time, does not affect the Lomb–Scargle algorithm. The derivation of the shifting parameter for the multivariate case is delineated for the time-discrete signal $\hat{S}$$=\{\left({\hat{S}}_{i},{\vec{t}}_{i}\right)\in R^{m+1},0\leq i\leq N-1\}.$ Starting with the orthogonality condition

$$\sum_{n=0}^{N-1}\sin\left({\vec{\omega }}_{k}\cdot {\vec{t}}_{n}-\tau_{k}^{*}\right)\cos\left({\vec{\omega }}_{k}\cdot {\vec{t}}_{n}-\tau_{k}^{*}\right)=0$$

and applying the trigonometric identities to remove the differences in the arguments, we have

$$\begin{array}{cc} \sum_{n=0}^{N-1}\left[\left(\underset{c_{n}}{\underset{︸}{\cos\left({\vec{\omega }}_{k}\cdot {\vec{t}}_{n}\right)}}\underset{c_{Ø}}{\underset{︸}{\cos\left(\tau_{k}^{*}\right)}}+\underset{s_{n}}{\underset{︸}{\sin\left({\vec{\omega }}_{k}\cdot {\vec{t}}_{n}\right)}}\underset{s_{Ø}}{\underset{︸}{\sin\left(\tau_{k}^{*}\right)}}\right)\right.\\ \left.\left(\underset{s_{n}}{\underset{︸}{\sin\left({\vec{\omega }}_{k}\cdot {\vec{t}}_{n}\right)}}\underset{c_{Ø}}{\underset{︸}{\cos\left(\tau_{k}^{*}\right)}}-\underset{c_{n}}{\underset{︸}{\cos\left({\vec{\omega }}_{k}\cdot {\vec{t}}_{n}\right)}}\underset{s_{Ø}}{\underset{︸}{\sin\left(\tau_{k}^{*}\right)}}\right)\right] & =0. \end{array}$$

By using the defined abbreviations and rearranging the summation (the terms $c_{Ø}$ and $s_{Ø}$ do not depend on *n*), the following equation is easily deduced:

$$\frac{c_{Ø}\;s_{Ø}}{{c_{Ø}}^{2}-{s_{Ø}}^{2}}=\frac{\sum_{n=0}^{N-1}c_{n}\;s_{n}}{\sum_{n=0}^{N-1}\left(c_{n}^{2}-s_{n}^{2}\right)}.$$

The fraction on both sides can be further simplified by applying $\cos^{2}\left(x\right)-\sin^{2}\left(x\right)=\cos\left(2x\right)$, $\cos\left(x\right)\sin\left(x\right)=\frac{1}{2}\sin\left(2x\right)$, and the definition of the tangent. This results in

(21) $$\begin{array}{cc} \tan(2\tau_{k}^{*}) & =\frac{\sum_{n=0}^{N-1}\sin\left(2{\vec{\omega }}_{k}\cdot {\vec{t}}_{n}\right)}{\sum_{n=0}^{N-1}\cos\left(2{\vec{\omega }}_{k}\cdot {\vec{t}}_{n}\right)}. \end{array}$$

Notice that in comparison with the one-dimensional LSM, the frequency $\omega_{k}$ is missing on the left side in Equation (21).

### 4.2. Parameter Estimation

Similar to the procedure for the LSM for a discrete signal in one dimension (see Equation (16)), a discrete multivariate signal $\left({\hat{S}}_{i},{\vec{t}}_{i}\right)$ with *N* samples is multiplied by $\cos({\vec{\omega }}_{k}\cdot \vec{t_{i}}-\tau_{k}^{*})$, resulting in

$$\begin{array}{c} \sum_{n=0}^{N-1}\hat{S}\left({\vec{t}}_{n}\right)\cos\left({\vec{\omega }}_{k}\cdot {\vec{t}}_{n}-\tau_{k}^{*}\right)=a_{k}\sum_{n=0}^{N-1}\cos^{2}\left({\vec{\omega }}_{k}\cdot {\vec{t}}_{n}-\tau_{k}^{*}\right)\\ \;+b_{k}\sum_{n=0}^{N-1}\underset{=0,\;\mathrm{if}\;\mathrm{orthogonal}}{\underset{︸}{\sin\left({\vec{\omega }}_{k}\cdot {\vec{t}}_{n}-\tau_{k}^{*}\right)\cos\left({\vec{\omega }}_{k}\cdot {\vec{t}}_{n}-\tau_{k}^{*}\right)}}+\sum_{n=0}^{N-1}\underset{\mathrm{random}\;\mathrm{error}}{\underset{︸}{\epsilon_{k}\left({\vec{t}}_{n}\right)\cos\left({\vec{\omega }}_{k}\cdot {\vec{t}}_{n}-\tau_{k}^{*}\right)}}. \end{array}$$

The determination of the parameter $a_{k}$ and its confidence interval $\Delta a_{k}$ are similar to the one-dimensional case shown in Equation (18):

$$\begin{array}{cc} \frac{\sum_{n=0}^{N-1}y\left({\vec{t}}_{n}\right)\cos\left({\vec{\omega }}_{k}\cdot {\vec{t}}_{n}-\tau_{k}^{*}\right)}{\sum_{n=0}^{N-1}\cos^{2}\left({\vec{\omega }}_{k}\cdot {\vec{t}}_{n}-\tau_{k}^{*}\right)}\; & =a_{k},\;\Delta a_{k}=\frac{4}{\pi }\Phi_{1-\alpha }\frac{\sigma_{0}}{\sqrt{N}}. \end{array}$$

The parameter $b_{k}$ and its confidence interval $\Delta b_{k}$ are calculated analogously:

$$\begin{array}{cc} \frac{\sum_{n=0}^{N-1}y\left({\vec{t}}_{n}\right)\sin\left({\vec{\omega }}_{k}\cdot {\vec{t}}_{n}-\tau_{k}^{*}\right)}{\sum_{n=0}^{N-1}\sin^{2}\left({\vec{\omega }}_{k}\cdot {\vec{t}}_{n}-\tau_{k}^{*}\right)}\; & =b_{k},\;\Delta b_{k}=\Delta a_{k}. \end{array}$$

We recall that the α-quantile of the sampling error distribution $\Phi_{1-\alpha }$ (with standard deviation σ) has been used to model the random error. The power spectral density, as well as the false alarm probability, are calculated in the same way as the one-dimensional LSM method (see the Supplementary Materials).

An implementation of the multivariate Lomb–Scargle method is available, in the spectral (R) package published on CRAN [24], to give the user access to a multidimensional analysis with an easy-to-use interface.

## 5. Application

The selected applications are ordered by the increasing complexity of the sampling and the data themselves. First, a regular grid with missing values for synthetic test data is considered. Second, Ultrasound Doppler Velocimetry (UDV) measurement data, which contain jitter and missing values, are analyzed. Finally, a general data scenario given by an astrophysical 2D set of sunspots, which appear freely in time and space, is investigated.

### 5.1. Synthetic Test Data

We start with a simple test case, where the input signal is a simple two-dimensional plain wave $z=\cos\left(2\pi \left(xf_{x}+yf_{y}\right)+\frac{\pi }{4}\right)$, with $f_{x}=3.25$ and $f_{y}=6.32$ representing the dimensionless frequencies. These fixed frequencies are selected in such a way that $f_{x},f_{y}\neq k\cdot \omega_{0}$, with $\omega_{0}=2\pi /T_{x,y}$ and $T_{x,y}=1$. This ensures the situation where the signal frequency does not map to any test frequency $k\cdot \omega_{0}$ if DFT is performed, which results in the worst-case scenario for all DFT-based methods. The aim is to enlarge the truncation error $\epsilon_{T}$ in case of the DFT for frequencies that do not match.

**Figure 2:**
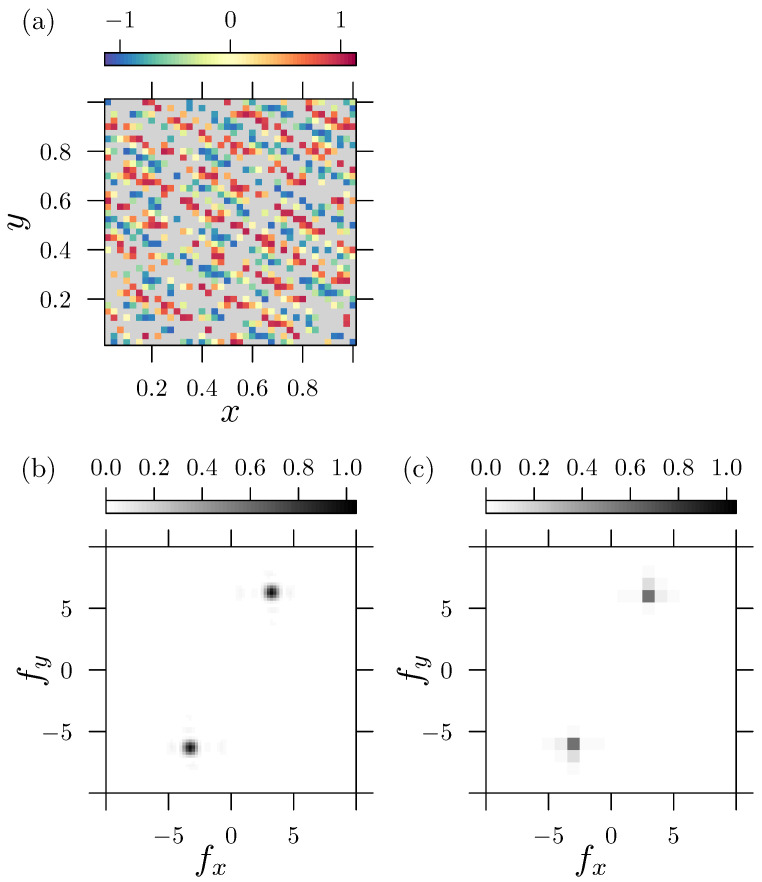
Comparison of the PSD calculated with the LSM and with the DFT approach for two-dimensional input data with missing values: (**a**) Sampled data $z=\cos(2\pi \left(xf_{x}+yf_{y}\right)+\pi /4)$, for x,y∈[−1,1] with sampling increment δx,δy=0.025, $f_{x}=3.25$, and $f_{y}=6.32$. Gray areas represent missing values. (**b**) PSD calculated with the LSM and (**c**) PSD calculated with the DFT. Notice that the truncation error and the sparse resolution leads to a rough approximation of the frequency and to a reduced amplitude (by 50%) in the PSD calculated using the DFT.

The 2D sampling of x,y∈[0,1] is performed with δx,δy=0.025. With respect to the chosen frequencies $f_{x}$ and $f_{y}$, it becomes evident that both frequencies do not fit to the data range by an integer fraction. Data gaps are introduced by removing randomly distributed and uncorrelated grid points covering 60% of the total dataset. The latter is an exaggerated example of how data with a high proportion of missing values can be analyzed using multivariate LSM. Therefore, Figure 2a shows the non-uniform distributed input data. Gray areas indicate missing values (non-available numbers “nan”). As the LSM requires an appropriate input vector of frequencies, we choose for both variables $f_{x},f_{y}\in \left[-10,10\right]$ with a resolution of $\delta f_{x},\delta f_{y}=0.025$. This ensures that the frequency space is sampled sufficiently densely.

The corresponding power spectral density (PSD) from the LSM (see the Supplementary Materials for the definition), shown in Figure 2b, displays the maxima at the first and third quadrants, which represent a wave traveling upwards. The figure illustrates that even with a lot of missing data, it is possible to properly detect the periodic signal. In the present case, the value of $\mathrm{psd}(f_{x},f_{y})\approx 1$ indicates a perfect fit to the corresponding sinusoidal model. For comparison, Figure 2c depicts the standardized PSD

$${\mathrm{psd}}_{\mathrm{FT}}\left(\vec{\omega }\right)=\frac{N}{N-1}\cdot \frac{1}{2\sigma_{0}^{2}}{\left(\frac{2\left|F\left(z(x,y)\right)\left(\vec{\omega }\right)\right|}{1-\frac{N_{\mathrm{zero}}}{N}}\right)}^{2}$$

calculated from a DFT denoted by the operator $F(z\left(x,y\right))\left(\vec{\omega }\right)=\int z\left(x,y\right)e^{i(\omega_{x}x+\omega_{y}y)}dx\;dy$. Here, missing values (nan) are handled by zero padding, which means that the introduced gaps are filled with zeroes. Since the DFT represents a conservative transformation (mapping) into the spectral domain, zero padding leads to a significant reduction in the average signal energy. A proper rescaling to the number of zeros $N_{\mathrm{zero}}$ (missing values) is required to ensure approximated amplitude estimations. Zero padding always changes the character of the input signal, in such a way that the corresponding DFT treats the zeros as if they were part of the original signal. The resulting PSD then becomes different from the original. Since the signal frequencies $f_{x},f_{y}$ do not fit into an integer-spaced scheme of the data range, the DFT suffers drawbacks, as briefly discussed in Section 2.2. The effects of leakage and the misfit of frequencies can be seen in Figure 2c, in terms of a rather coarse resolution and a lower PSD value.

### 5.2. Three-Dimensional UDV Flow Measurement

**Figure 3:**
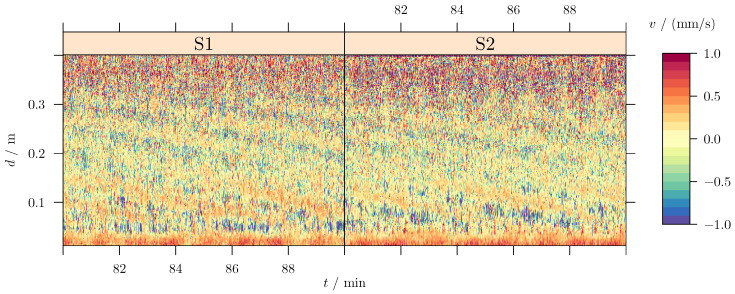
Sensor data in the co-rotating reference frame. Data are taken as a time series from two rotating sensors. The measurement time of the sensor data is folded with the individual phase location; so, $\varphi_{n}^{S_{1}}=\omega_{\mathrm{out}}t_{n}\;(\mathrm{sensor1},\;S1)$ and $\varphi_{n}^{S_{2}}=\varphi_{n}^{S_{1}}+\pi \;(\mathrm{sensor2},\;S2)$ describe the two ordinates.

The second example is taken from previous experimental flow measurements [26] devoted to investigating the azimuthal magneto-rotational instability (AMRI). The experiment consists of a cylindrical annulus containing a liquid metal between the inner and the outer wall. The inner wall rotates at a frequency $\omega_{\mathrm{in}}=2\pi \cdot 0.05\;\mathrm{Hz}$, whereas the outer wall rotates at a frequency $\omega_{\mathrm{out}}=2\pi \cdot 0.013\;\mathrm{Hz}$. The flow is driven by the shear, since $\omega_{\mathrm{in}}-\omega_{\mathrm{out}}>0$. Two ultrasound sensors mounted on the outer cylinder, on opposite sides of each other and at the same radius, measure the axial velocity component $v_{z}$ along the line of sight parallel to the rotation axis of the cylinder. Additionally, the liquid metal flow is exposed to a magnetic field $B_{\varphi }\propto r^{-1}$ (*r* is the radial coordinate), originating from a current $I_{\mathrm{axis}}$ on the axis of the cylinder. A typical time series of the axial velocity (along the measuring line) is displayed in Figure 3. The existence of periodic patterns (large blue inclined stripes) in this figure indicates a traveling wave propagating in the fluid. In cylindrical geometry, a traveling wave is described in terms of the drift frequency ω=2πf, the vertical wave numbers *k*, and the azimuthal wave numbers *m*. A close look at Figure 3 shows that this wave is not axially symmetric, and the leading azimuthal wave number is m=1. This is indeed proven in the following through the LSM analysis.

The measured time series for $v_{z}$ depends on the time $t_{n}$, depth $d_{n}$, and the angular positions of the sensors $\varphi_{n}^{S_{1}}=\omega_{\mathrm{out}}t_{n}$ and $\varphi_{n}^{S_{2}}=\varphi_{n}^{S_{1}}+\pi$, where the subscript *n* indicates the sampling. The azimuthal angles depend on the time, since the sensors are attached to the outer wall. This induces the mapping $v_{z}=f\left(t_{n},d_{n},\varphi_{n}\right)$, where $\varphi_{n}=\left\{\varphi_{n}^{S_{1}},\varphi_{n}^{S_{2}}\right\}$ is meant to be one of the two sensors at each sampling instance. Finally, the measured velocity component is a mapping $v_{z}:R^{3}\mapsto R$, which fits perfectly to the proposed multivariate LSM.

**Figure 4:**
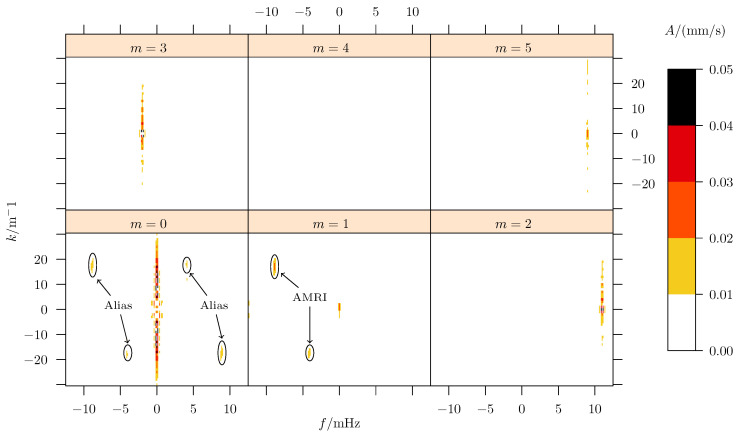
Spectrum of sensor data. The amplitude spectrum is calculated from the data in Figure 3 with the LSM. The weak alias peaks in m=0 panel originate from the sensor mismatch and aliasing effect due to the outer rotation. The latter acts as a sampling frequency and, therefore, projects the AMRI wave to m=0.

Figure 4 illustrates the result of the LSM decomposition into several azimuthal *m*-modes with the corresponding amplitudes. The m=0 mode contains a stationary structure, at a frequency f≈0, which originates from sensor mis-alignments and (thermal) side effects in the flow. The minor non-stationary components, here the two point-symmetric peaks, originate from crosstalk (or alias projections, see labels on Figure 4) of the m=1 mode. This is mainly for two reasons: First, the sensors do not behave identically (e.g., misalignment or different sensitivity) and respond slightly differently to the same flow signal. This means that one of the sensors projects a little more energy into the data than the other, which leads to a “leakage” effect and a weak signal in the m=0 mode. Second, the rotation of the sensors acts as an additional sampling frequency $f_{\mathrm{out}}$. In consequence, the spectrum of this regular sampling function folds with the spectrum of the observed process, leading to alias images (copies) of the original process spectrum into other frequency ranges (i.e., *m*s). In the present multivariate analysis, these alias structures exhibit point symmetry in the m=0 panel, indicating corresponding traveling waves. It is evident that these aliases originate as a systematic error from the LSM method and, due to their symmetry, effectively cancel each other out. One could also argue that the multivariate LSM enables a robust identification of alias amplitudes that do not belong to the observed process.

The AMRI wave itself is located in the m=1 panel, with a characteristic frequency in time (*f*) and in the vertical direction (*k*). Here, two components can be identified: the dominant wave at f≈9mHz and $k\approx 20\;m^{-1}$ and a minor counterpart at f≈4mHz and $k\approx -20\;m^{-1}$. The signals observed in the m=3 and m=5 panels might be due to aliases because of the rotation of the inner cylinder at a frequency $f_{i}=0.05\;\mathrm{Hz}$.

The advantage of “high” dimensional spectral decomposition is the improved noise rejection. With respect to the raw data, given in Figure 3, it is obvious that high-frequency noise is present in the data. Depending on the exact distribution of this noise, its energies spread over a certain range of frequencies. If we could select a representative depth and angle so that $d_{n},\varphi_{n}=\mathrm{const}.$, the velocity $v_{z}$ would only depend on time. In the subsequent one-dimensional analysis, the noise would accumulate along the single frequency ordinate, probably hiding the signal of interest. Taking the higher-order analysis distributes the noise energies over multiple domain variables. For the present example, this means that the distortions are projected into higher frequencies *f*, higher *m*s, and larger *k*s. Since the signal of interest remains in the same spectral corridor, its signal amplitude becomes clearer because of the “reduced” local noise.

### 5.3. Analyzing 2D Sunspot Data

**Figure 5:**
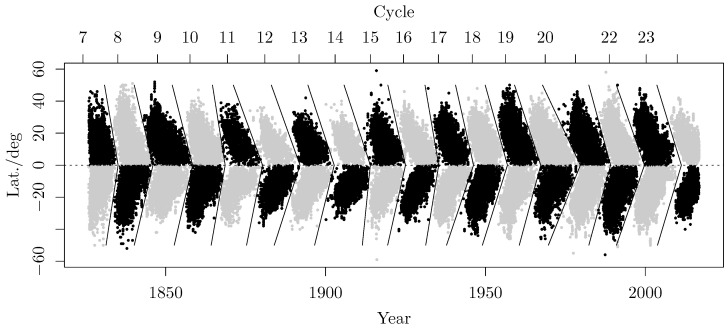
Butterfly diagram of the sunspot data. The separation of the individual wings took place according to the procedure presented in Leussu et al. [43]. The colored patches indicate the changing polarity of each cycle. The tilted segmentation lines depict the optimized borders between two cycles.

Since the beginning of the 17th century, systematic visual observations of sunspots have been available. This enables the investigation of dynamic processes taking place in the sun. The appearance of sunspots on the sun’s surface depends on the level of solar activity; therefore, it renders some fundamental features of the underlying solar dynamo, such as the 11yr solar cycle. Since the beginning of the 1820s, observational data have been available, in terms of a two-dimensional time series, as displayed in Figure 5. The data exhibit a periodic wing-like pattern, essentially symmetric with respect to the sun’s equator, forming the sunspot butterfly diagram. This diagram summarizes the individual sunspot groups appearing at a certain time and latitude on the Sun, for the past 190 years. Additionally, Figure 5 depicts the assigned field polarity order indicated by the sunspot color (gray or black). Given a year *Y* and a mean latitude *L* (degrees), the field polarity P(Y,L)∈{−1,1} gives the arrangement of the leading north/south polarity of a sunspot group. This polarity is changing approximately every 11 years, which is called Schwabe’s cycle. The full period of 22years is called the Hale cycle. The segmentation, shown in Figure 5, was obtained following the suggestions from Leussu et al. [43], with some simplifications leading only to minor mis-assignments for individual sunspot groups.

The sunspot data are taken “as-is” from Leussu et al. [44], which originate from several sources with different levels of quality, i.e., the Royal Greenwich Observatory–USAF/NOAA(SOON) (Available at http://solarscience.msfc.nasa.gov/greenwch.shtml, (accessed on 10 October 2025)) [45,46], Schwabe [47], and Spoerer [48] datasets. The reader might also refer to the historic publications of Spoerer [49], Spoerer and Maunder [50]. A detailed discussion of the data collection is given by Leussu et al. [43,44] and the referenced literature.

In this section, the LSM spectral decomposition from this unevenly sampled binary dataset is obtained in order to identify typical periods. In contrast to the two examples analyzed in Section 5.1 and Section 5.2, the sunspot dataset consists of real, arbitrary sampling in both time and space, providing the most general scenario input data for the LSM. The starting point for the subsequent analysis is the simplified segmentation of the butterfly diagram, reassigning the field polarity. The segmentation takes place by optimizing the distance *d* (in the (L,Y)-plane) between each sunspot and the segmentation line

$$L\left(Y\right)=\left\{\begin{array}{cc} m_{1}\left(Y-T_{0}\right) & m_{1}<0,L>0\\ m_{2}\left(Y-T_{0}\right) & m_{2}>0,L\leq 0 \end{array}\right.,$$

which is a piecewise linear function with intersection point $\left(T_{0},0\right)$. The individual slopes, with $|m_{1}\left|,\right|m_{2}|\;<12\;\deg/\mathrm{yrs}$, are assigned on the northern and southern hemispheres, respectively. The final segmentation, shown in Figure 5 consists of 18 individual cycles subdivided by a “>”-shaped separation area.

The LSM spectral decomposition is based on the definition of a two-dimensional wave model

(22) $$P\left(Y,L\right)∼A\cos\left(\omega_{Y}Y+\omega_{L}L+\tau^{*}\right)+B\sin\left(\omega_{Y}Y+\omega_{L}L+\tau^{*}\right)+\epsilon ,$$

where *P* is given by the assigned patch polarity. To show the advantage and robustness of the LSM, the raw data are employed without any further preprocessing. The selected frequencies $\omega_{Y,n},\omega_{L,n}\propto n^{-1}$ are given on a rectangular grid with inverse distance so that the periods are distributed uniformly alongside the consecutive counter *n*. Furthermore, the frequency resolution close to $|\omega_{L}|=0$ is selected as finer to better resolve this region.

**Figure 6:**
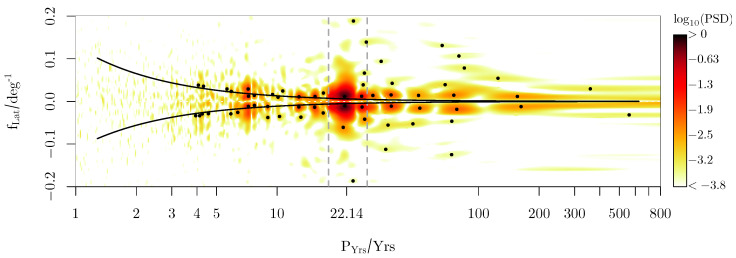
Two-dimensional LSM spectrum of the sunspot data. The curved line follows the path line $v={\left(f_{L}P_{Y}\right)}^{-1}=\mathrm{const}.$, indicating a wave structure with a certain time dependence. The range of the solar cycle period is given by the vertical dashed gray lines.

Figure 6 gives the resulting LSM spectral decomposition in a reciprocal log-scale plot to analyze the different periods. Each of the selected peaks (numbered black dots) provides an FAP value of $p<10^{-10}$, meaning that these periods are significantly different from the noise level. Each of these points originates from a local frequency refinement to achieve the local maximum amplitude. The binary order information {−1,1} induced by the sunspots introduces higher harmonics into the spectrum, which also appears with a significantly low FAP value.

Figure 6 provides good agreement between the peaks found and the commonly known periods. Table 1 summarizes the major outcome in comparison with the literature. For example, the 22year Hale cycle varies from 18 to 28years (Usoskin [51]), which is identified by the two main peaks. Since the solar cycle is modulated—we refer the reader to Hathaway [52]—it is natural that a broad spectrum with many harmonics is present. These subsequent patterns are related to a set of local maxima, mainly collapsing on a horizontal line at $\left|f_{\mathrm{Lat}}\right|\approx 1.4\cdot 10^{-2}\;\deg^{-1}$, indicating that the complex structure of the individual wing shapes of the butterfly diagram with different widths, heights, and orientations corresponds to a main period $P_{\mathrm{Hale}}=21.634\;\mathrm{yrs}$. Next, we can identify typical periods, which are related to the Gleissberg process (see Table 1), or the short periods in the range of P≈7.2yrs, which are consistent with the data provided by Prestes et al. [53] and Kane [54] and partly with Deng et al. [55].

The latitudinal dimension of the spectrum spreads the peaks vertically, which is an advantage over a purely 1D analysis, where all signals would be projected on the $f_{\mathrm{Lat}}=0$ line. In addition, complex patterns such as the lines of constant phase velocities (dashed curve of Figure 6) may be used to analyze modulations in the dynamics of the sunspot motion.

## 6. Conclusions

In the present work, a multidimensional extension of the Lomb–Scargle method was developed. The key aspect is the redefinition of the phase argument to ${\vec{\phi }}_{\mathrm{new}}=\vec{\omega }\cdot \vec{t}-\tau^{*}$. We suggest using a modified shifting parameter $\tau^{*}$ in contrast to the traditional approach, which shifts the ordinate $\phi_{\mathrm{orig}}=\omega \cdot \left(t-\tau \right)$ instead of the phase. This enables multivariate modeling with a single scalar value $\tau^{*}$ for all independent variables, as there is always a shifting parameter, $\tau^{*}$, for which $\int_{a}^{b}\sin\left(\phi \left(t\right)\right)\cos\left(\phi \left(t\right)\right)\;\;dt$ vanishes on any interval [a,b].

With respect to the evaluation of the measurement results, the noise rejection $\epsilon_{\mathrm{LS}}\propto 4N^{-0.5}/\pi$ and confidence intervals ($\Delta a_{k}$ and $\Delta b_{k}$) of the model parameters $a_{k}$ and $b_{k}$ are delineated. To emphasize the advantages of the LSM, it is compared with the traditional Fourier mode decomposition. The systematic error $\epsilon_{T}$ does not vanish for FT-based methods with the increasing number of samples on a fixed interval *T*, which finally leads to the common leakage effect. Here, the signal amplitudes are distributed on an area of neighboring spectral components; i.e., individual bins or pixels. We conclude that the standard orthogonal mode-related procedures do not represent a consistent estimator for model parameters $a_{k}$ and $b_{k}$, whereas the introduction of $\tau^{*}$ in the LSM leads to a consistent estimator with $\epsilon_{T}=0$, even for higher dimensions. Finally, it was shown that the LSM converges to the true model parameters with an increasing number of samples and provides better noise rejection ($\epsilon_{\mathrm{LS}}<\epsilon_{\mathrm{FS}}$) as well.

The examples from Section 5 underline the strengths of the developed procedure in a consecutive way. First, the application on ideal two-dimensional test data shows the ability to analyze fragmented time series. Here, the sampling remains regular, meaning $t=nT_{0}$ with n∈N, but with $n_{i}-n_{i+1}\neq 1$ as an incomplete set of locations to describe the missing values. A second quasi-similar situation is given in the experimental dataset of UDV measurements, for which a certain level of jitter is expected. The dimensionality in this example was extended to $R^{3}$ to decompose the wave parameters in frequency *f*, symmetry *m*, and spatial frequency *k*.

As the third analysis, sunspot datasets represent the most general case of uneven sampling. Taking only the time series of positions into account, the LSM is able to calculate the spectrum on an individual frequency grid, pronouncing the low frequencies close to zero. The assignment of {−1,1} to each data point is a minor modification, such that the data can be assumed to be binary raw data. The spectral decomposition with the LSM shows the characteristic footprint of waves and its dynamics present in the butterfly diagram. The time–frequency spectrum itself provides the commonly known frequencies, i.e., Hale Cycle or Gleissberg process. The second frequency $f_{\mathrm{Lat}}$ provides information about the minor peaks, which spread into the latitudinal domain. Moreover, from these values, the sunspot drift motion may be calculated using a characteristic *v*-line with its side bands.

## Figures and Tables

**Table 1:** Common peaks. Period values are given in years. Values above 100yrs are affected by the low period resolution caused by the limited time span of sunspot data. The results from Prestes et al. [53] refer to the Schwabe cycle and, therefore, are doubled.

Process	Common Period	From Spectrum	
Eddy	515	559	(1)
	350	359	(1)
Hale (Schwabe)	22.14 (11.07)	21.63 ± 2.5	(2)
	18…28		(2)
Gleissberg	88 (80…150)	78, 85, 125, 156	(1), (2)
–	126	125	(3)
–	2 × 3.6	7.2	(4)
–	2 × 3.9	7.7	(4), (5)

(1) McCracken et al. [56]; (2) Usoskin [51]; (3) Ogurtsov et al. [57]; (4) Prestes et al. [53] (Figure 6); (5) Kolotkov et al. [58].

## Data Availability

The data required for this study are either cited or included in the Supplementary Materials section of this publication.

## Supplementary Materials

The following supporting information can be downloaded at https://www.mdpi.com/article/10.3390/s25216535/s1, (i) All R-scripts calculating the presented Figures, (ii) raw data set and processed data set from the MRI experiment at HZDR, (iii) sun spot data and processed data set including tabulated significant frequencies from the sun spot data set. References [59,60,61] is cited in the Supplementary Materials.
