# Supplementary material for: Multivariate Frequency and Amplitude Estimation for Unevenly Sampled Data Using and Extending the Lomb–Scargle Method
Source: Sensors (Basel). 2025 Oct 23;25(21):6535. doi: 10.3390/s25216535 (PMC12608236; doi:10.3390/s25216535)
Supplement: Supplementary file 1 [file sensors-25-06535-s001.zip › sensors-3892943-supplementary.pdf]

# Supplementary Materials: Multivariate Frequency and Amplitude Estimation for Unevenly Sampled Data Using and Extending the Lomb–Scargle Method

**Martin Seilmayer [1,*,†], Thomas Wondrak [2,†] and Ferran Garcia [3,†]**

## 1. Definition of the coefficients $a_k$

In comparison with the classical definition of Lomb [1], the coefficients $a_k$ and $b_k$ differ by a factor of $\sqrt{N/2}$, scaling the result. With a closer look at the original equations

$$a_{k,\mathrm{orig}} = \frac{\sum_n \hat{s}_n \cos(\omega_k(t_n - \tau_k))}{\sqrt{\frac{N}{2}}\sqrt{\sum_n \cos^2(\omega_k(t_n - \tau_k))}}$$

this factor becomes evident, e. g. Hocke [2]. Since $\lim_{n\to\infty} \sum_n \cos^2(\omega_k(t_n - \tau_k)) \approx N/2$ can be assumed, $a_k \approx a_{k,\mathrm{orig}}$ is valid, if $N$ fits exactly to a multiple of $\omega_k$. Because the presented approach is related to the rather technical amplitude demodulation procedure, we assume equation (18) of the main manuscript to be more accurate.

## 2. OMD as a non-consistent estimator

In the following, only signals $s(t) \in \mathbb{R}$ described in terms of a finite set of individual frequency components $\omega_k, 0 \le k \le M$ $(k \in \mathbb{N}^+)$, are considered. Furthermore, band limitation is assumed so that there exists an upper maximum frequency $\omega_k < \omega_{\max}$.

The analysis is based on the trigonometric model definition from equation (1) of the main manuscript, with the coefficients $a_k, b_k \in \mathbb{R}$. The model misfit $\epsilon(t)$ is defined by the difference between the observed signal $s(t)$ and the assumed model function $y(t)$ as seen in equation (2) of the main manuscript. Then, the signal

$$s(t) = \sum_{k=0}^{M} (a_k \cos(\omega_k t) + b_k \sin(\omega_k t) + \epsilon_k(t))$$

is a sum of a trigonometric models with $M$ coefficients and individual error values. The total model misfit $\epsilon(t) = \sum_k \epsilon_k(t)$ originates form measurement uncertainties with unknown distribution.

However, the sine and cosine functions are assumed to be an orthonormal basis, which is valid for the infinite integral, as seen in Section 2.2. To shorten the explanations, the following derivation refers only to the cosine term and neglects the corresponding sine term, which can be derived in a similar manner.

To show the effects of OMD applied to discrete sampled functions, e. g. time series from measurements, the next passage derives the sampling series and its discrete model representation.

In general, a continuous signal can be described as a function defined for every $t \in \mathbb{R}$. But a realistic measurement or observation $y(t)$ of such a process takes place in the range from $t = 0$ to arbitrary time $t = T$. Therefore, we associate to the measurement a windowed signal $s_\mathrm{w}(t)$ defined for every $t \in \mathbb{R}$ as

$$s_\mathrm{w}(t) = \big(y(t) + \epsilon(t)\big)w(t) \quad \text{with } w(t) = \begin{cases} 1 & 0 \le t < T \\ 0 & \text{otherwise} \end{cases}$$

where the sampling error $\epsilon(t)$ is included. The window function $w(t)$ ensures the finite observation time $0 \le t < T$ but leave the infinite definition range untouched.

The sampling procedure, described in the next step, relies on the Dirac distribution and its properties. The Dirac impulse is defined as

$$\delta(t) = \begin{cases} \infty & t = 0 \\ 0 & t \neq 0 \end{cases} \text{ with } \int_{-\infty}^{\infty} \delta(t)\,\mathrm{d}t = 1.$$

The so-called sifting property

$$\int_{-\infty}^{\infty} \delta(t-\tau)\cdot\phi(t)\,\mathrm{d}t = \phi(\tau), \tag{S1}$$

can be expressed for every function $\phi(t)$. Using this identity the convolution function

$$\Psi(t) = \sum_{n=-\infty}^{\infty} \delta(t - nT_\mathrm{s})$$

helps to describe the sampling of the signal $s_\mathrm{w}(t)$ with sampling rate $T_\mathrm{s}$.

The continuous description of the sampling series

$$A(t) = s_\mathrm{w}(t)\Psi(t)$$

mathematically models the sampling which takes place while taking a time series measurement of a physical process.

According to the theory of orthogonal function decomposition (e. g. Cohen [3, Chap. 15]), the individual model coefficients of equation (1) of the main manuscript

$$a_k = \frac{2}{T}\int_{-\infty}^{\infty} y(t)\cos(\omega_k t)\,\mathrm{d}t \quad \text{and} \quad b_k = \frac{2}{T}\int_{-\infty}^{\infty} y(t)\sin(\omega_k t)\,\mathrm{d}t \tag{S2}$$

can be recovered by integrating over the model function.

In order to estimate the coefficients $a_k, b_k$, the integration of Eq. (S2) has to be carried out on the sampled series $A(t)$ representing the acquired data. Assuming that the trigonometric model $y(t)$ approximates the sampling series $A(t)$ in the limit $\epsilon(t) \to 0$:

$$\begin{aligned} a_k &= \frac{2}{T}\int_{-\infty}^{\infty} y(t)\cos(\omega_k t)\,\mathrm{d}t \approx \frac{2}{T}\int_{-\infty}^{\infty} A(t)\cos(\omega_k t)\,\mathrm{d}t = \\ &= \frac{2}{T}\int_{-\infty}^{\infty} \big(y(t)+\epsilon(t)\big)w(t)\sum_{n=-\infty}^{\infty}\delta(t-nT_\mathrm{s})\cos(\omega_k t)\,\mathrm{d}t, \end{aligned}$$

and by factorizing and exchanging the sum with the integral

$$\begin{aligned} a_k &= \frac{2}{T}\sum_{n=-\infty}^{\infty}\int_{-\infty}^{\infty} \underbrace{\big(y(t)+\epsilon(t)\big)w(t)\cos(\omega_k t)\delta(t-nT_\mathrm{s})}_{\text{sifting property (S1)}}\,\mathrm{d}t = \\ &= \frac{2}{T}\sum_{n=-\infty}^{\infty}\big(y(nT_\mathrm{s})+\epsilon(nT_\mathrm{s})\big)w(nT_\mathrm{s})\cos(\omega_k nT_\mathrm{s}), \end{aligned} \tag{S3}$$

where the applied sifting property of the Dirac distribution achieves the sampling at discrete time instances $nT_\mathrm{s}$. Due the definition of the rectangular window function $w(t)$, the sum

with $nT_s < 0$ and $nT_s > T$ is exactly zero, which leads to a finite summation range. With the identity of the total number of samples taken, $N = T/T_s$, the coefficient $a_k$ reads

$$a_k = \frac{2}{T}\sum_{n=0}^{N-1}\big(y(nT_s) + \epsilon(nT_s)\big)\cos(\omega_k nT_s) =$$
$$= \frac{2}{T}\sum_{n=0}^{N-1}\left(\sum_{j=0}^{M}\big(a_j\cos(\omega_j nT_s) + b_j\sin(\omega_j nT_s) + \epsilon(nT_s)\big)\right)\cos(\omega_k nT_s)$$

Figure (1) of the main manuscript sketches the scenario with $T_s = f_s^{-1}$ as the sampling period and $T > 2\pi/\omega_0$. With respect to the integrals of Eq. (S2), the dashed right area causes errors in two ways, when quadrature demodulation for the OMD is carried out. First, the energy of that amplitude is spread into the next neighboring integer $k$s and second, the truncation error (dashed area) causes a mismatch of $a_k$ (and $b_k$) which only depends on $T$ but *not* on the amount of sampling points $N$ used.

In the following steps, the coefficients $k \neq j$ are neglected, since they are projected on the error $\epsilon(t)$. The remaining $k$-th set of parameters $k = j$ is sufficient to derive the four statements (i)-(iv) of Section (2.2) of the main manuscript.

### *2.1. Properties of the truncation error*

Considering the Fourier decomposition of the sampling series $A(t)$:

$$\int_0^T \sum_{n=0}^{N-1} s_w(nT_s)\delta(t - nT_s)\cos(\omega_k nT_s)\,\mathrm{d}t = \int_0^T \delta(t - nT_s)\,\mathrm{d}t\, S_N$$
$$S_N = \sum_{n=0}^{N-1} a_k\cos^2(\omega_k nT_s) + b_k\sin(\omega_k nT_s)\cos(\omega_k nT_s) + \epsilon_k\cos(\omega_k nT_s). \quad \text{(S4)}$$

The latter enables the limitation of the integration and summation boundaries. Next, by applying the sifting property (S1), the continuous time series becomes independent from time so that

$$\sum_{n=0}^{N-1} s_w(nT_s)\cos(\omega_k nT_s) = S_N \quad \text{(S5)}$$

describes the measured (sampled) data points at time instances $nT_s$ of the signal. The expression above represents the well known sum of the $k$-th cosine term of discrete Fourier series. The corresponding sine term is defined in a similar manner.

By employing the trigonometric identities $\cos^2(x) = \frac{1}{2}(1 + \cos(2x))$ and with $\sin(x)\cos(x) = \frac{1}{2}\sin(2x)$ to the definition of $S_N$ given in Eq. (S4), and multiplying by $2/N$, Eq. (S5) becomes

$$\frac{2}{N}\sum_{n=0}^{N-1} s_w(nT_s)\cos(\omega_k nT_s) =$$
$$= a_k\left(1 + \frac{1}{N}\sum_{n=0}^{N-1}\left[\cos(2\omega_k nT_s) + \frac{b_k}{a_k}\sin(2\omega_k nT_s)\right]\right)$$
$$+ \frac{2}{N}\sum_{n=0}^{N-1}\epsilon_k\cos(\omega_k nT_s) \quad \text{(S6)}$$

Assuming $b_k = 0$ for a pure cosine signal, the truncation error $\epsilon_k^{\mathrm{T}}$ defined as

$$\epsilon_k^{\mathrm{T}} = \frac{1}{N}\sum_{n=0}^{N-1}\cos(2\omega_k nT_s)$$

can be divided into two components

$$\epsilon_k^{\mathrm{T}} = \frac{1}{N}\left(\underbrace{\sum_{n=0}^{N_{2\pi}} \cos(2\omega_k n T_\mathrm{s})}_{=0} + \sum_{n=N_{2\pi}+1}^{N-1} \cos(2\omega_k n T_\mathrm{s})\right),$$

from which the first vanishes because it covers an integer number of periods $\omega_k$. The right one consequently describes the truncation error with respect to a $2\omega_k$ frequency as given in the gray area in Figure (1) of the main manuscript.

A Taylor series decomposition gives

$$\begin{aligned}\epsilon_k^{\mathrm{T}} &= \frac{T_\mathrm{s}}{T}\sum_{n=N_{2\pi}+1}^{N-1}\left(1 - \frac{1}{2!}(2\omega_k n T_\mathrm{s})^2 + \underbrace{\frac{1}{4!}(2\omega_k n T_\mathrm{s})^4 - \dots}_{\ll 1}\right) \\ &\approx \frac{T_\mathrm{s}}{T}\left(\Delta N - 2(\omega_k T_\mathrm{s})^2 \sum_{n=N_{2\pi}}^{N-1} n^2\right),\end{aligned}$$

with $\Delta N = N - N_{2\pi}$. This is further reduced with $\sum_{n=1}^{N} n^2 = \frac{N(N+1)(2N+1)}{6}$ and $T_\mathrm{s} = T/N$

$$\begin{aligned}\epsilon_k^{\mathrm{T}} &\approx \frac{\Delta N}{N} - 2(\omega_k T)^2 \frac{\Delta N(\Delta N+1)(2\Delta N+1)}{6N^3} \\ &\approx \frac{\Delta N}{N}\left(1 - 2(\omega_k T)^2\left(\frac{2\Delta N^2}{6N^2} + \underbrace{\frac{3}{6N^2} + \frac{1}{6N^2\Delta N}}_{\to 0}\right)\right) \\ &\approx \frac{\Delta N}{N}\left(1 - \frac{2}{3}(\omega_k T)^2\left(\frac{\Delta N}{N}\right)^2\right) \approx \frac{\Delta\varphi}{T} \le 0.2 \qquad \text{(S7)}\end{aligned}$$

so that $\epsilon_k^{\mathrm{T}}$ becomes independent of the sampling rate $T_\mathrm{s}$. The "time phase" $\Delta\varphi = \min_i(T - \pi i/\omega_k)$, $i \in \mathbb{N}$ covers the range marked as gray area in Figure (1) of the main manuscript. The maximum value of $\epsilon_k^{\mathrm{T}} \le 0.2$ originates from the $\cos(2\omega_k n T_\mathrm{s})$-term if only one period plus truncation fits into the integration window. Moreover equation (S7) implies that a higher sampling frequency – which gathers more information from the process – will not lead to a more precise approximation of $a_k$ (and of course $b_k$). Therefore, the quadrature demodulation (or even the Fourier series decomposition) is *not* a consistent estimator for amplitude and phase, because it will not converge

$$\lim_{N\to\infty} \sum_{n=0}^{N-1} s_\mathrm{w}(nT_\mathrm{s}) \cos(\omega_k n T_\mathrm{s}) \neq a_k$$

towards the "true" $a_k$ for a given finite $T$. Instead, it converges in the limit of $T \to \infty$.

### *2.2. Confidence Intervals of Model Parameters – (iii)*

The last term of Eq. S6 corresponds to the sampling error $\epsilon_k^{\mathrm{FS}}$, which might be encountered in real measurements. Given a normal distributed error function $\epsilon_k = \mathcal{N}(0, \sigma)$ with

$\Phi_{1-\alpha}$ as the corresponding $\alpha$-quantile of the error distribution related to the underlying process with its standard deviation $\sigma$ (e. g. JCGM [4]), it turns out that

$$\epsilon_k^{\mathrm{FS}} = \frac{2}{N}\sum_{n=0}^{N-1} \epsilon_k \cos(\omega_k n T_\mathrm{s}) = \frac{2}{N}\sum_{n=0}^{N-1} \mathcal{N}(0,\sigma)\cos(\omega_k n T_\mathrm{s}) =$$
$$= \frac{2}{N}\Phi_{1-\alpha}\frac{\sigma}{\sqrt{N}}\sum_{n=0}^{N-1}\cos(\omega_k n T_\mathrm{s}) < \Phi_{1-\alpha}\frac{2\sigma}{\sqrt{N}} \quad \text{(S8)}$$

which vanishes in the limit $N \to \infty$. This is valid because a linear combination of normally distributed variables keeps normally distributed (e. g. Parzen [5, Theorem 4A, p. 90]). In addition, expression (S8) gives the upper limit of the parameter confidence interval.

In summary, the estimation of Fourier coefficients $a_k$ (and $b_k$)

$$\frac{2}{N}\sum_{n=0}^{N-1} s_\mathrm{w}(nT_\mathrm{s})\cos(\omega_k n T_\mathrm{s}) \approx a_k\left(1 + \frac{\Delta\varphi}{T}\right) \pm \Phi_{1-\alpha}\frac{2\sigma}{\sqrt{N}}$$

is affected by the truncation error with respect to the full period of $\omega_k$ and a random error from the measurement. The first is independent from the sampling which proves that Fourier decomposition is a non consistent estimator for amplitude and frequency. The last term, the random error, converges to zero in the limit of large $N$, as expected.

## 3. Power Spectral Density and False Alarm Probability

In this work, the simplest case of uncorrelated and mean free Gaussian noise is assumed which suits many common technical and scientific cases. From the power spectral density (PSD) $P_k = \frac{N}{4\sigma_0^2}(a_k^2 + b_k^2)$, with $\sigma_0^2 = \frac{1}{N-1}\sum_{n=0}^{N-1}(y(t_n) - \bar{y})^2$ as the variance of the sample, refer to Hocke [2] and Zechmeister and Kürster [8], the standardized PSD is defined by

$$\mathrm{psd}(\omega_k) = P_k p_k$$

on the interval $[0,1]$, where $p_k$ is the standardized Gaussian noise. Here, $P_k$ is similar to a signal to noise ratio given in equation (19) of the main manuscript (see Scargle [6]). Since LSM calculates the result of a least square fit, a value of $\mathrm{psd}(\omega_k) = 1$ indicates a “perfect” fit to the corresponding model function. In the case of $\mathrm{psd}(\omega_k) = 0$, no correspondence is visible. The discussion about the presented standardization is carried out in detail by Cumming et al. [7]. The different ways to perform the calculation of the psd-value are briefly summarized in Zechmeister and Kürster [8]. Additionally, a more precise description of noise takes some effort which should be accomplished by analyzing the measurement data or by taking additional noise measurements. The different procedures are briefly described by Cumming et al. [7] and Horne and Baliunas [9].

The standardized noise level reads $p_k = 2/(N-1)$, so the standardized power spectral density

$$\mathrm{psd}(\omega_k) = \frac{N}{N-1}\frac{A_k^2(\omega_k)}{2\sigma_0^2} \quad \text{(S9)}$$

can be calculated directly from the power spectral density or amplitude. A more sophisticated approach relies on a Bayesian estimate of $\mathrm{psd}(\omega_k)$ which is presented in Mortier et al. [10]. For most technical applications, equation (S9) should be sufficient.

As a statistical measure, the probability

$$\mathcal{P}(P_k > P_0) = (1 - \mathrm{psd}(\omega_k))^{\frac{N-3}{2}}$$

states that there is no PSD peak $P_k$ larger than a reference value $P_0$ of the best fit. From here, the statistical significance of a single frequency $\omega_k$ can be deduced as the so called false alarm probability (FAP) with

$$\text{FAP} = \begin{cases} 1 - (1 - \mathcal{P}(P_k > P_0))^M, & \text{if } \mathcal{P}(P_k > P_0) \approx 1 \\ M\mathcal{P}(P_k > P_0), & \text{if } \mathcal{P}(P_k > P_0) \ll 1 \end{cases}, \tag{S10}$$

where $M$ denotes the number of independent (fundamental) frequencies present in the signal. The discussion about this degree of freedom is very diverse in literature and is discussed for instance by VanderPlas [11]. The first approach is Shannon's sampling theorem as a pragmatic and conservative access to this topic. It states that the number of independent frequencies is $M \approx N/2$. At the same moment a band limited signal is required which is sampled with twice the maximum signal frequency, i. e. $f_\text{s} \geq 2f_\text{max}$. It follows that signal frequencies above $f_\text{s}/2$ become visible as an alias in the lower frequency domain. In this aspect, randomly sampled data may behave different. For randomly sampled data the conservative approach defines an average sampling rate $\overline{f_s} = N/T$ which will lead to $M \approx T\overline{f_s}/2$ as a lower limit. The parameter $T$ scales the total sampling (e. g. time) range interval in one dimension. However, the question about the possible maximum frequency that can be detected in randomly sampled data still remains. If we assume sampling points originating from a regular grid, but with randomly distributed missing values, then $f_s \approx \min(\Delta t)^{-1}$ relates to the minimal distance between two neighboring points as upper limit of $f_\text{s}$. Data in such a grid is taken at $t_i = t_1 + n_i p$ instances, where $p$ is a kind of a common divisor, refer to Eyer and Bartholdi [12] and $n_i$ is a non complete set of values to reach every location. Given $n_i \in \mathbb{N}^+$ we will find that the effective maximum frequency fulfills $f_s > \overline{f_s}$ . Care must be taken with this assumption, because it could lead to undesired large values of $f_s$ and therefore wrong estimations of $M$.

Horne and Baliunas [9] carried out an extensive study about the number of independent frequencies (and the maximum detectable frequency). They found an empirical approximation

$$M = -6.362 + 1.193N + 0.00098N^2,$$

which is a compromise between the conservative $N/2$ and the artificially large minimal distance value. A detailed discussion on FAP and the independent frequencies can be found in the studies by Baluev [13,14,15].

## References


1. Lomb, N.R. Least-Squares Frequency Analysis of Unequally Spaced Data. *Astrophysics and Space Science* **1976**, *39*, 447–462. https://doi.org/10.1007/BF00648343.
2. Hocke, K. Phase Estimation with the Lomb-Scargle Periodogram Method. *Annales Geophysicae* **1998**, *16*, 356–358.
3. Cohen, L. *Time-Frequency Analysis*; Prentice Hall signal processing series, Prentice Hall PTR: Englewood Cliffs, N.J, 1995.
4. JCGM. *Evaluation of measurement data – Guide to the expression of uncertainty in measurement*; Joint Committee for Guides in Metrology, Application Note JCGM 100:2008 E, 2008.
5. Parzen, E. *Stochastic Processes*; Holden Day Series in Probability and Statistics, Holden-Day: San Francisco, 1962.
6. Scargle, J.D. Studies in Astronomical Time Series Analysis. II - Statistical Aspects of Spectral Analysis of Unevenly Spaced Data. *The Astrophysical Journal* **1982**, *263*, 835–853. https://doi.org/10.1086/160554.
7. Cumming, A.; Marcy, G.W.; Butler, R.P. The Lick Planet Search: Detectability and Mass Thresholds. *The Astrophysical Journal* **1999**, *526*, 890. https://doi.org/10.1086/308020.
8. Zechmeister, M.; Kürster, M. The Generalised Lomb-Scargle Periodogram. A New Formalism for the Floating-Mean And Keplerian Periodograms. *Astron. Astrophys.* **2009**, *496*, 577–584. https://doi.org/10.1051/0004-6361:200811296.
9. Horne, J.H.; Baliunas, S.L. A Prescription for Period Analysis of Unevenly Sampled Time Series. *The Astrophysical Journal* **1986**, *302*, 757. https://doi.org/10.1086/164037.

10. Mortier, A.; Faria, J.P.; Correia, C.M.; Santerne, A.; Santos, N.C. BGLS: A Bayesian formalism for the generalised Lomb-Scargle periodogram. *Astronomy & Astrophysics* **2015**, *573*, A101. https://doi.org/10.1051/0004-6361/201424908.
11. VanderPlas, J.T. Understanding the Lomb-Scargle Periodogram. *The Astrophysical Journal* **2017**, *236*.
12. Eyer, L.; Bartholdi, P. Variable Stars: Which Nyquist Frequency? *Astronomy and Astrophysics Supplement Series* **1999**, *135*, 1–3. https://doi.org/10.1051/aas:1999102.
13. Baluev, R.V. Assessing the Statistical Significance of Periodogram Peaks. *Monthly Notices of the Royal Astronomical Society* **2008**, *385*, 1279–1285.
14. Baluev, R.V. Detecting Multiple Periodicities in Observational Data with the Multifrequency Periodogram—II. Frequency Decomposer, a Parallelized Time-Series Analysis Algorithm. *Astronomy and Computing* **2013**, *3-4*, 50–57. https://doi.org/10.1016/j.ascom.2013.11.003.
15. Baluev, R.V. Detecting Multiple Periodicities in Observational Data with the Multifrequency Periodogram – I. Analytic Assessment of the Statistical Significance. *Monthly Notices of the Royal Astronomical Society* **2013**, *436*, 807–818. https://doi.org/10.1093/mnras/stt1617.


**Disclaimer/Publisher's Note:** The statements, opinions and data contained in all publications are solely those of the individual author(s) and contributor(s) and not of MDPI and/or the editor(s). MDPI and/or the editor(s) disclaim responsibility for any injury to people or property resulting from any ideas, methods, instructions or products referred to in the content.
