# Supplementary figures and images for: Multivariate Frequency and Amplitude Estimation for Unevenly Sampled Data Using and Extending the Lomb–Scargle Method
Source: Sensors (Basel). 2025 Oct 23;25(21):6535. doi: 10.3390/s25216535 (PMC12608236; doi:10.3390/s25216535)

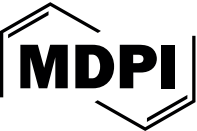

Supplement: Supplementary file 1 [file sensors-25-06535-s001.zip › Definitions/EpsToPdf.pdf]

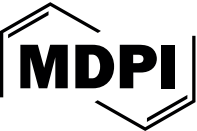

Supplement: Supplementary file 1 [file sensors-25-06535-s001.zip › Definitions/logo-mdpi-eps-converted-to.pdf]

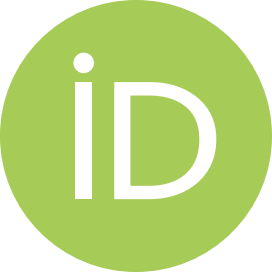

Supplement: Supplementary file 1 [file sensors-25-06535-s001.zip › Definitions/logo-orcid.pdf]

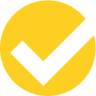

check for  
updates

Supplement: Supplementary file 1 [file sensors-25-06535-s001.zip › Definitions/logo-updates-eps-converted-to.pdf]

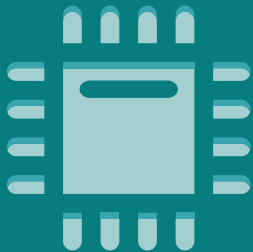

*sensors*

Supplement: Supplementary file 1 [file sensors-25-06535-s001.zip › Definitions/sensors-logo-eps-converted-to.pdf]

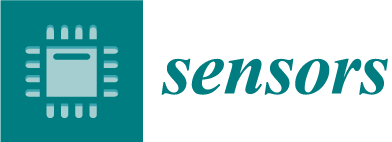

Supplement: Supplementary file 1 [file sensors-25-06535-s001.zip › Definitions/sensors-logo.png]
